# Impact of Nanomaterials on the Regulation of Gene Expression and Metabolomics of Plants under Salt Stress

**DOI:** 10.3390/plants11050691

**Published:** 2022-03-03

**Authors:** Zainul Abideen, Maria Hanif, Neelma Munir, Brent L. Nielsen

**Affiliations:** 1 Dr. Muhammad Ajmal Khan Institute of Sustainable Halophyte Utilization, University of Karachi, Karachi 75270, Pakistan; zuabideen@uok.edu.pk; 2 Department of Biotechnology, Lahore College for Women University, Lahore 54000, Pakistan; mariahanif81@yahoo.com; 3 Department of Microbiology and Molecular Biology, Brigham Young University, Provo, UT 84602, USA

**Keywords:** salinity, ecophysiology, environment, salt tolerance, photosynthesis

## Abstract

Plant salinity resistance results from a combination of responses at the physiological, molecular, cellular, and metabolic levels. This article focuses on plant stress tolerance mechanisms for controlling ion homeostasis, stress signaling, hormone metabolism, anti-oxidative enzymes, and osmotic balance after nanoparticle applications. Nanoparticles are used as an emerging tool to stimulate specific biochemical reactions related to plant ecophysiological output because of their small size, increased surface area and absorption rate, efficient catalysis of reactions, and adequate reactive sites. Regulated ecophysiological control in saline environments could play a crucial role in plant growth promotion and survival of plants under suboptimal conditions. Plant biologists are seeking to develop a broad profile of genes and proteins that contribute to plant salt resistance. These plant metabolic profiles can be developed due to advancements in genomic, proteomic, metabolomic, and transcriptomic techniques. In order to quantify plant stress responses, transmembrane ion transport, sensors and receptors in signaling transduction, and metabolites involved in the energy supply require thorough study. In addition, more research is needed on the plant salinity stress response based on molecular interactions in response to nanoparticle treatment. The application of nanoparticles as an aspect of genetic engineering for the generation of salt-tolerant plants is a promising area of research. This review article addresses the use of nanoparticles in plant breeding and genetic engineering techniques to develop salt-tolerant crops.

## 1. Introduction

Soil salinization of land poses a serious threat and harms the environment, agriculture, and the economy. Salinity stress in plants may cause changes at the molecular as well as the physiological level [1]. Some plants contain salt tolerance genes while many have a salt-sensitive genetic makeup. Various complex mechanisms may alter the genetic responses in plants under abiotic conditions. Modifications in the expression of salt-responsive genes make the plants more resistant to salinity stress. Ecophysiological traits of plants and their importance for biomass production in response to variable climate change are critical for sustainable agricultural productivity [2,3,4]. Plants can change their ecophysiological mechanism in five known constraints including growth, water dynamics, mineral nutrition, photosynthesis rate, and oxidative stability [5,6]. 

The adaptation of a plant to a stressful environment is a complex and sensitive phenomenon [7,8]. This acclimation is governed by multiple genes and regulatory pathways [9]. Once the plant detects a stress, it first senses and then transduces a stress signal. Plants utilize various components for signal transduction including transcription factors, ion transporters, kinases, calcium, and hormones [10]. During abiotic stress, many physical modifications occur such as alteration in protein and other metabolites along with changes in the cellular matrix and segregation of nucleic acid strands [11]. All these alterations may result in altered regulation of abiotic stress-responsive genes. It was observed by Tang [12] that superoxide dismutase is responsible for oxidative stress tolerance. Enhanced salt resistance in plants is due in part to the overexpression of chloroplast protein-increasing stress tolerance (CEST) [13]. The assimilation of methylglyoxal in a saline stressed potato plant was inhibited by glyoxalase activity [14]. Hasanuzzaman et al. [15] reported that selenium protects plants from damaging free radicals, improves the antioxidant defense system, and methylglyoxal detoxification. It was observed that the use of selenium nanoparticles with bitter melon induced alterations in the methylation of cytosine in DNA resulting in epigenetic modifications. The up-regulation of the *WRKY1* transcription factor was induced by a high dose of selenium nanoparticles. The transcription of phenylalanine ammonia-lyase (*PAL*) and 4-CoA-ligase (4CL) genes have also been affected by selenium nanoparticles [10].

The application of nanoparticles to plants helps to mitigate salinity stress. Nanoparticles can be used to alter plant genetic makeup to become resistant to salt stress. Nanoparticles are identified as particles that have a size of less than 100 nm in diameter [16]. They are found naturally in various resources such as minerals or as a product of bacteria and clays. Nanoparticles have been used historically for coloring metals and other purposes, with new applications over the past several years [17]. Nanoparticles that are engineered have some significant specific properties. These nanoparticles have different sizes and shapes and their composition also varies, and they differ widely from naturally occurring nanoparticles [18]. Metal and metal oxide nanoparticles reveal various physiochemical properties such as high density and possess microscopic edges on their surface. The sizes of nanoparticles vary due to differences in composition, such as Cu^+2^O, Zn^+2^O^−2^, Sn^+4^O^−2^_2_, Al^+3^_2_O^−2^_3_, Mg^+2^O^−2^, Ti^+4^O^−2^_2_, and Ce^+4^O^−2^_2_. Due to the changes in nanoparticles size, many properties including magnetic, electronic, and chemical properties are altered. Magnetic nanoparticles have achieved significant importance due to their variations in size and shape [19]. Surface, optical, thermal, and electrical properties can also be incorporated into these nanoparticles. The process of metal/metal oxide nanoparticle synthesis includes the reduction as well as oxidation of respective metal salts [20]. There are many different factors that contribute to nanoparticle reactivity with desired biomaterials. These factors are the size, dimension, and stability of the nanoparticles [21]. In the past few decades, synthesized nanoparticles have been used for various industrial and household purposes. There is continuing effort to synthesize new nanomaterials to enhance quality products. However, the environment can be contaminated due to the excess use of nanoparticles due to improper disposal of industrial wastes and other by-products [22].

Nanoparticles can be adapted for environmental conditions and their aggregation and oxidation state can be engineered [23]. The stability and behavior of nanoparticles can be affected by chemicals in the environment and by physical parameters. The properties of nanoparticles depend on their composition. The composition of nanoparticles also affects their rate of reaction, penetration ability, and translocation inside the plant. Hence, the same nanoparticles may show different responses in plants under different conditions. For instance, it was observed by Barrios et al. [24] that plant responses were influenced by citric acid-coated nanoparticles compared to bare nanoparticles. Plants constantly interact with the surrounding medium, such as water, air, and soil. The engineered nanoparticles can cause different effects caused by quantum dots, carbon-based and metal-based effects on plant growth variations, physiological and biochemical traits, food production, and quality of food. Thorough interaction studies between engineered nanoparticles and plants are needed to analyze the toxicity levels and the remediation scheme to build a sustainable environment for agriculture [25]. Plants play a significant part in the ecosystem and in the food chain. However, the effects of nanoparticles on plants are not well known. The study of nanoparticles is difficult due to a lack of detection methods in plants [26]. The most suitable technique for the identification of nanoparticles in plants is inductively coupled plasma mass spectroscopy (ICP-MS). Due to the size, shape, composition, and stability of nanoparticles, the plant may show positive or negative impacts due to nanoparticle application. Several reported studies showed that some nanoparticles have a negative impact on plants such as declines in plant growth, production rate, and pigments [27]. Conversely, some nanoparticles may be beneficial for plants. In order to maintain their stability in agricultural crop production, synthetic nanoparticles are mostly used. These nanoparticles are used as biofertilizers, growth stimulators, soil-improving agents, and are also used as sensors [28].

## 2. Engineered Nanoparticles and their Effect on Plant Salt Tolerance Genes: Enzymatic Expression

Engineered nanoparticles can interact chemically and mechanically with plants. These interactions are based on their properties such as size, surface area, and catalytic interactions. Few studies have been reported regarding the effect of nanoparticles at the molecular level [29,30,31]. Various plant species are highly affected by ZnO nanoparticles. Nanoparticles penetrate the plant leaf and accumulate in the edible parts while some assimilate into the soil in the surrounding area of the plant. Some metal and metal oxide nanoparticles are toxic to the environment, such as Ag^+1^, Fe^+3^, Zn^+2^, Al^+3,^ and Ti^+4^ [32]. It was observed that when *Brassica juncea* was treated with silver nanoparticles it resulted in increased levels of antioxidant enzymes, for instance, guaiacol peroxidase, catalase, and ascorbate peroxidase, which resulted in decreased levels of reactive oxygen species (ROS) activity [33]. The activity of enzymes such as super oxide dismutase, catalase, guaiacol peroxidase, ascorbate peroxidase, and glutathione reductase increased after the treatment of *Brassica juncea* with gold nanoparticles [34]. It was found that H_2_O_2_ and proline content increases in gold nanoparticle-treated plants. The activity of ascorbate peroxidase, glutathione reductase, and guaiacol peroxidase is stimulated in the presence of up to 400 ppm of gold nanoparticles, while on the other hand, the activity of guaiacol peroxidase increases with 200 ppm gold nanoparticles. Plant molecular responses to silver nanoparticle treatment were analyzed in *Aradidopsis* by reverse transcription-polymerase chain reaction [35]. A whole-genome cDNA expression microarray was also used for the transcriptional response analysis of *Arabidopsis* plants subjected to silver nanoparticles. This resulted in the identification of 286 upregulated genes, including those involved with metal and oxidative stress responses such as the vacuolar proton exchanger, SOD, cytochrome P450-dependent oxidase, and peroxidase. It also identified about 81 downregulated genes along with genes that help in the plant defense system. These included auxin-regulated genes, ethylene signaling pathway, and SAR against pathogens.

**Figure 1:**
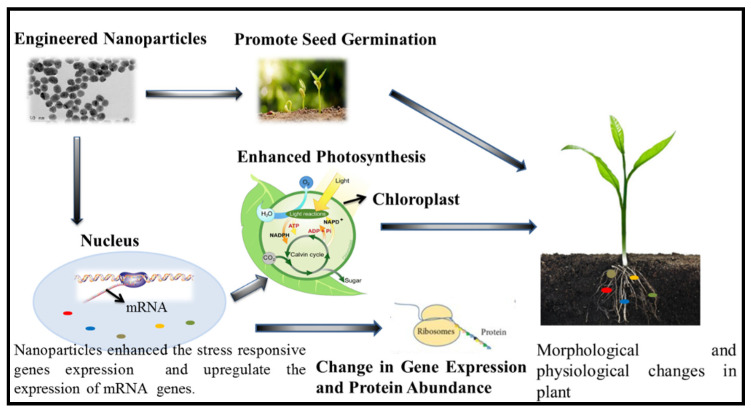
Effects of engineered nanoparticles on plant ecophysiological mechanisms in response to salinity.

A proteomic analysis of rice treated with silver nanoparticles was carried out. It was found that silver nanoparticle-responsive proteins were associated with various metabolic functions such as transcription and protein degradation, the oxidative stress response pathway, and the calcium signaling pathway [36]. Treatment with zinc oxide nanoparticles in *Arabidopsis thaliana* identified 660 up- and 826 down-regulated genes. Seedling growth and seed germination of tomato was enhanced by the up-regulation of stress-related gene expression employing multi-walled carbon nanotube-based treatment [37]. The effect of engineered nanoparticles on plant traits is shown in Figure 1.

Iron (Fe) is considered to be essential for plant growth and development as it plays a significant role in enzymatic reactions, helps in photosynthesis, and aids to improve the performance of photosystems. In plants, Fe is present in the insoluble form, i.e., Fe^3+^. The increase in pH and aerobic conditions leads to a decreased concentration of Fe in the soil. The use of iron nanoparticles helps to improve plant resistance to different environmental abiotic stresses. The application of iron nanoparticles reacts at the molecular level of plants, which helps to enhance the nutrient uptake ability [38]. Toxicity in plants may be caused by an excess concentration of iron nanoparticles. A higher amount of free Fe ions such as Fe^2+^ and Fe^3+^ leads to the production of ROS in plants. It was reported by Rodríguez et al. [39] that in some plants, down-regulation of detoxifying proteins such as CAT2 (CATALASE 2; AT4G35090) protein and AP2 (PEROXIDASE 2; AT5G06720) protein has been observed. A deficiency of Fe in the roots of *M. truncatula*, *P. dulcis,* and *P. persica* was correlated with superoxide dismutase expression, i.e., ATMSD1 (ARABIDOPSIS-SIS-MANGANESE SUPEROXIDE DISMUTASE 1; AT3G10920) [39]. Fe deficiency may cause the production of non-enzymatic ROS. Under Fe deficiency in *A. thaliana,* two enzymes have been reported to be expressed: GST1 (ARABIDOPSIS GLUTATHIONE S-TRANSFERASE 1; AT1G02930) and MDAR1 (MONODEHYDROASCORBATE REDUCTASE 1; AT3G52880) [40]. The ROS-eliminating enzyme aids in the stimulation of the ascorbate-glutathione cycle from GPX3 (GLUTATHIONE PEROXIDASE 3; AT2G43350) [40]. Due to the magnetic properties of superparamagnetic iron oxides, Fe_2_O_3_ (maghemite) and Fe_3_O_4_ (magnetite) nanoparticles are widely used in various applications including the mitigation of salinity effects of plants. High Fe_3_O_4_ nanoparticle concentration has a high impact on seed germination and root elongation of cucumber [41]. In cucurbits, the Fe_3_O_4_ nanoparticle aggregation occurred in the stem and roots [42]. The toxicity of superparamagnetic iron oxide nanoparticles has been tested in *Lemna gibba* [43]. It has been observed that plant chlorophyll content decreased while the photosynthetic activity and growth were also highly affected. The size and stability of nanoparticles are responsible for their toxicity level. The effect of Fe_3_O_4_ nanoparticles has been investigated in *Cucumis sativus,* and it was observed that seed germination and root elongation were highly affected [41]. It has been shown that Fe_3_O_4_ nanoparticles are translocated towards the foliage, stem, and below-ground root. Aggregation of Fe_3_O_4_ nanoparticles in plants may decrease the root hydraulic movement and water transport. The growth parameters of *S. lycopersicum* were studied by the application of Fe_2_O_3_ nanoparticles. It has been observed that these nanoparticles were clogged in root hairs, root tips, and the nodal portion of plants. Increases in Fe_2_O_3_ nanoparticle concentration improved iron content in plants [44]. In *Arachis hypogaea,* root length and plant height increased due to the use of Fe_2_O_3_ nanoparticles in saline conditions [45].

While a number of genes with the potential for the engineering of salt tolerance have been identified and tested, additional genes and regulatory pathways need to be identified. Work in many labs is ongoing to develop genomic, transcriptomic, proteomic, and metabolomic resources.

## 3. Plant Metabolomics and the Linkage of Molecular Functions to Nanomaterial Application

**Figure 2:**
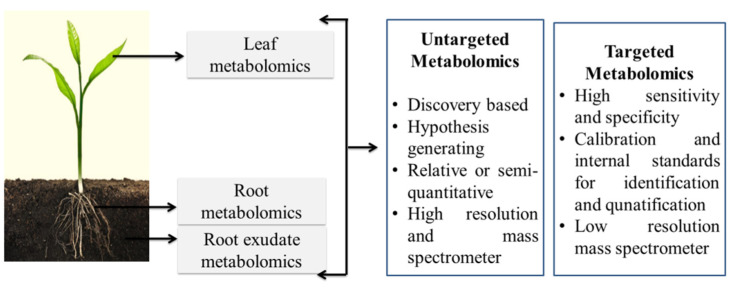
Metabolomics analysis in plants exposed to engineered nanomaterials.

The by-products of cellular regulatory mechanisms are metabolites. These metabolites are secreted in response to the external stimuli faced by the organism. More than 200,000 metabolites are secreted by plants and these metabolites are divided into two classes; these are primary and secondary metabolites for plant growth and development [46]. Primary metabolites are essential and include carbohydrates, fatty acids, vitamins, amino acids, and organic acids [47]. Polyketides, alkaloids, terpenoids, glucosinolates, and phenylpropanoids are secondary metabolites synthesized from primary metabolites and are required by plants for adaptation and defense responses [48]. Throughout the plant kingdom, primary metabolites are common in all plants and conserved in their structure, while on the other hand, plant secondary compounds may vary in their chemical composition and are species-specific. Figure 2 shows the metabolomics analysis of plants exposed to engineered nanomaterials. In xenobiotic plants, the modifications in plant physiology induced by engineered nanoparticles are monitored by molecular events. These molecular events also reflect the metabolites that participate in biological pathways, for instance, the citric acid cycle, glycolysis, gluconeogenesis, and amino acid and secondary metabolite biosynthesis, nitrogen, and fatty acid metabolism. In order to defend against or adapt to various abiotic stresses, plant roots excrete metabolites as signaling molecules. Plants also alter soil chemistry and biochemical pathways to enhance nutrient bioavailability [49]. A list of halophytic species and their secondary metabolites is shown in Table 1.

## 4. Plant Genetic Responses to Salinity Stress

Under salt stress, genetic responses in plants occur by a complex mechanism. The synthesis of specific gene products (RNA or protein) is up-regulated while others are down-regulated. It was observed that these mechanisms may occur at different phases of the central dogma process, such as transcription initiation, RNA processing, post-transcriptional processing, translation, and modification [51]. In order to identify the genes responsible for the stress response, transcriptional profiling is most often used, leading to the creation of extensive databases. To date, considerable information is available on the transcription factors that are included in the up- and down-regulation of plant genes and salt-responsive genes [52]. These genomic methodologies play a vital function in the encoding, cloning, and characterization of salt-responsive genes. These factors are considered important for the up-regulation and down-regulation of gene expression. *bZIP, MYB, WRKY, AP2, C2H2* zinc finger gene, *NAC,* and *DREB* family proteins are stress-responsive gene family members. The cis-acting binding of a transcription factor at a promoter region can alter the expression of individual genes [53].

Under salinity stress, up-regulation in *bZIP* gene expression was observed in a salt-sensitive variety of wheat while in the salt-tolerant cultivar, down-regulation of the gene occurred [54]. Rice contains transcriptional regulators that play a vital function under stress responses. These regulators are *DREB1/CBF, DREB2,* and *AREB/ABF* [55]. Under salt stress, up-regulation of transcription factors (*OsNAC5* and *ZFP179*) occurs. The up-regulation of these factors may affect the synthesis of proline, LEA proteins, and sugar, which in turn plays a significant role in abiotic stress tolerance [56]. Zhang et al. [57] determined that one member of one gene family, i.e., the *MYB* gene, responds to abiotic stress. Sixty full-length cDNA sequences were isolated that encode wheat *MYB* proteins. The phylogenetic tree that includes wheat, rice, and Arabidopsis *MYB* proteins was used to analyze their function and evolutionary relationship. Up-regulation of *AtWRKY8* gene expression occurs in *Arabidopsis* plants under salinity stress [58]. In diverse species of plants, many genes and transcription factors are up-regulated under salinity stress as presented in Table 2 [59], which shows associated genes, molecular chaperones, and dehydration-related transcription factors. For ion homeostasis, the *SOS* gene family plays a vital function in salt tolerance [60]. It was observed by Schmidt et al. [61] that more than 10 genes involved in the osmotic regulation process are up-regulated in *Spartia alterniflora* under salinity stress.

**Figure 3:**
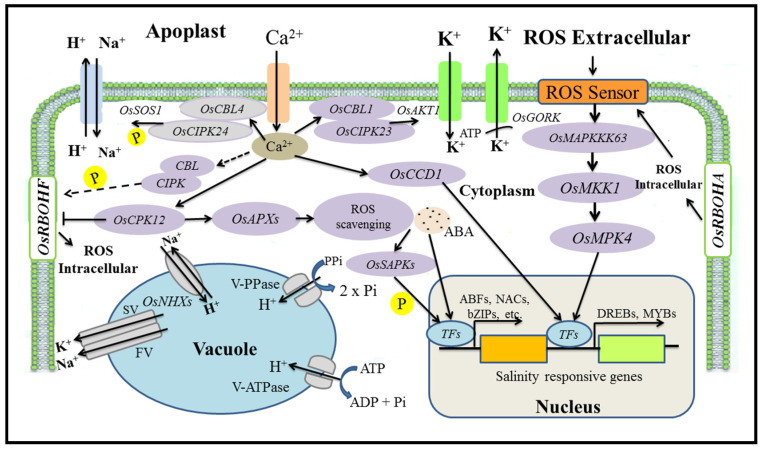
Signaling pathways in rice under salt stress.

The salt-responsive transcription factor *ERF1* (*SERF1)* gene was characterized by Schmidt et al. [61] in *Oryza sativa*. It was observed that salt treatment and H_2_O_2_ may induce the expression of this gene in roots. Plants lacking the transcription factor *SERF1* are less resistant to salinity stress than wild-type plants. However, the overexpression of *SERF1* may improve salt resistance in plants. Kinases play a vital role in the adaptation of plants to salinity stress and they also regulate the activity of transcription factors. In rice, *OsRMC* acts as a negative regulator during salinity stress and it also encodes a receptor-like kinase [62]. Negative gene expression was also shown by two transcription factors, *OsEREBP1* and *OsEREB*, which belong to the *AP2/ERF* family. As with the *OsRMC* promoter, the above-mentioned transcription factors bind to the same GCC-like DNA motif. One of the major transcription factors, the basic region/leucine zipper (*bZIP*), contains a specific region that binds to the DNA and a leucine zipper dimerization motif. One of the major *ABRE*-binding transcription factors, *OSBZ8,* was also recognized in rice and is identified as the most expressed gene in salt-tolerant cultivars [63]. To respond to environmental stresses such as abiotic stress, the alternative splicing of RNA/mRNA plays a significant role by switching on/off transcriptional activities. The spliceosomal proteins play a significant function in plant development, response to harsh environmental factors, the plant immune system, and tolerance efficiency [64]. Figure 3 summarizes the signaling pathway in rice under salt stress.

**Figure 4:**
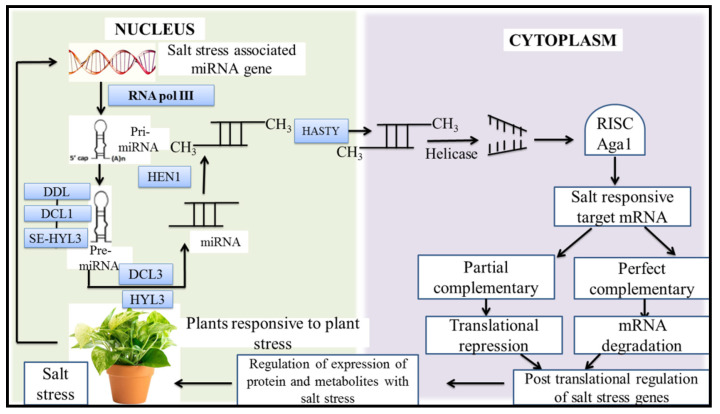
Pathway showing post-transcriptional regulation of salt stress-responsive plant genes mediated by miRNA.

MicroRNAs and small interfering RNA (siRNAs) could play a significant role in the abiotic stress responses of plants. However, post-transcriptional gene regulation has a significant impact on plant salinity resistance [78]. It has been observed that miRNAs are sensitive to biotic as well as abiotic stress. Plants face various environmental abiotic stresses such as drought, salinity, and cold. The overexpression of miR402 is induced by these environmental stresses. Figure 4 shows the post-transcriptional regulation of plant salt stress-responsive genes mediated by miRNA as studied in *Arabidopsis thaliana* and *Oryza sativa* to determine the significant role of miRNA in salt stress [79]. 

## 5. Mechanisms for the Regulation of Salt Tolerance Genes

Plant transcription factors act to link salt-sensitive pathways to several tolerance responses. Certain transcription factor family genes are expressed specifically in response to external salinity stress. These transcription factor gene families include *bZIP*, *WRKY, APETALA2*/Ethylene Response Factor (*AP2/ERF*), *MYB*, basic helix-loop-helix (*bHLH*), and NAC. These transcription factors play a vital function in regulating the expression level of genes that may enhance the plant salt tolerance level [80]. Salt stress-induced *bZIP* transcription factor *bZIP24* is a primary example involved in adaptation to salt resistance in plants. In *Arabidopsis,* this transcription factor induces the expression of many stress-responsive genes [81]. Salt tolerance is increased in *Arabidopsis* due to the expression of *bZIP24*. It was observed that *bZIP24* down-regulated *AtHKT1;1* directly or indirectly [82]. *bZIP24* was identified by comparison of transcript regulation patterns in *Arabidopsis* and in the halotolerant *Lobularia maritima.* This halophilic model species can be utilized for the identification of novel salt tolerance mechanisms [83].

### 5.1. Salinity Tolerance Mechanisms

*AtHKT1;1* regulates the sodium level in leaves and salt tolerance in plants. In order to modify a Na^+^-resistant crop, the molecular mechanism regulating *AtHKT1;1* expression should be determined [84]. The plant salinity tolerance mechanism was analyzed by the study of the model plant *Arabidopsis* [85]. *Rice transcription factor SALT-RESPONSE ERF1 (SERF1)* functions as an enhancer of ROS-activated MAP kinase under salinity stress. The high salinity rate may induce the expression of *SERF1.* It was observed that rice plants deficient in *SERF1* exhibit a drop in salt stress tolerance genes. *serf1* mutants grown hydroponically for 3-4 weeks were observed to be salt-sensitive while *SERF1*-overexpression lines showed increased salt tolerance. The Na^+^ over K^+^ ratio in the foliage of the *serf1* mutant was higher than the wild-type [86].

### 5.2. Traditional Plant Breeding

Many approaches including conventional plant breeding have been used to enhance the salinity resistance of plants that are economically important. These traditional breeding programs have been successful in improving salt stress tolerance [87]. However, multiple salt-responsive genes are involved in tolerance to salinity in plants. These genes also function with other parts of stress signal transduction pathways. The results obtained by traditional plant breeding may not identify genomic regions such as in perennial quantitative trait loci (QTL) that control salt resistance. By the improvement of high-throughput genotyping methods, molecular marker technology was developed to identify QTL, which is considered to be accountable for salt tolerance. In breeding programs, the selection efficiency is based on the identification of QTL regions [88]. The results of gene expression analysis and the correlation of QTLs may be easily detected at the seedling stage as compared to the developmental stages, while at the reproductive stages they are fully recognizable [89]. In recent studies, the antioxidant response in tomato plants under salinity stress was identified. The QTLs related to antioxidant content were also analyzed. The development of tomato cultivars having higher antioxidant levels may be accomplished using QTLs [90].

By using traditional breeding methods, multiple traits have been introduced into crop plants to manipulate salt tolerance. In addition, the overexpression of single genes can be used for improving plant salt tolerance. High salt tolerance has also been observed in genetically modified plants by the overexpression of genes that code for the synthesis and assimilation of osmoprotectants (proline and glycine betaine are used for osmotic adjustment). In addition, some enzymes such as glutathione S-transferase, peroxidase, ascorbate peroxidase, superoxide dismutase, and glutathione reductase are also used to modify plant salt resistance and oxidative protection [91]. Plant genetic engineering holds great promise for producing salt-tolerant crops. Salinity tolerance is multi-genic in nature, but most genetically engineered plants possess a single transgene. Various genetic tools are developed that can be utilized in gene function analysis. In order to develop salt-tolerant cultivars, the use of nanoparticle carriers to facilitate genetic engineering will be helpful to understand the mechanisms and regulation of gene expression, candidate gene usage, and tissue-specific and inducible promoters.

## 6. Salt Responsive Genes Present in Halophytes

Limited literature is available on the molecular defense mechanism of halophytes against salinity stress. In various studies, the ecological, physiological, anatomical, and biochemical responses of halophytes towards salinity were studied [92,93]. For the investigation of salinity stress defense molecular mechanisms, *Arabidopsis thaliana* was used as a model plant. This species was also utilized for the analysis of salt stress-responsive genes to enhance salinity tolerance in genetically modified plants [94]. It is generally understood that halophytes are salt-tolerant and glycophytes are salt sensitive. However, there are some halophyte species that are sensitive to abiotic stresses and some glycophytes are tolerant to salt. Halophytes are considered to be suitable model plants for the analysis of salt tolerance mechanisms [95]. It was observed that almost all plants likely possess similar salt tolerance regulatory mechanisms [96].

**Figure 5:**
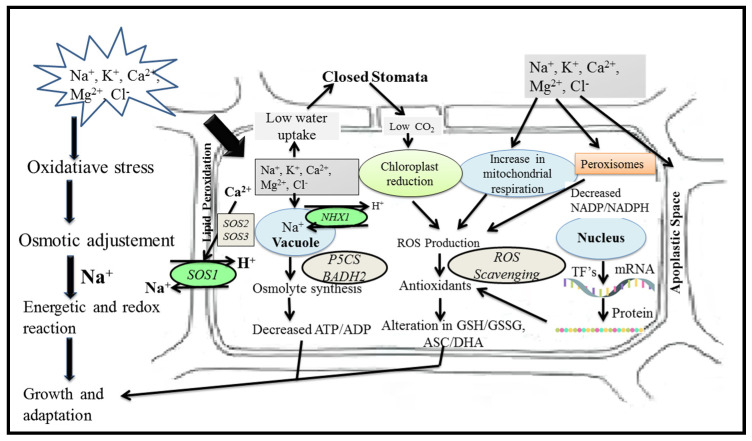
Salt stress tolerance mechanisms of halophytes in a saline environment.

Many genomic methods have been used for the isolation and identification of salinity-responsive genes from halophytes. The overexpression of these halophytic genes under the control of the constitutive, non-specific 35SCaMV promoter in glycophytic recipients may increase abiotic stress tolerance. Many of these genes code for Na^+^/H^+^ antiporters, vacuolar pyrophosphatase, potassium transporters, ion channels, antioxidants, and ROS scavengers. These genes also code for proteins that are included in signal transduction and various protective functions. Many other salt-responsive genes have been identified from halophytes such as *Salicornia brachiata* [97]. Many model plants including *Arabidopsis thaliana* and salt-tolerant *Thellungiella salsuginea* were used for the study of abiotic stress tolerance. *Thellungiella* exhibits high tolerance to salt and drought stress [98]. The genetic basis of the abiotic stress defense mechanism was obtained from the analysis of the genomic sequence of *Thellungiella salsuginea.* This species was identified as the gene resource for cation transporters, abscisic acid signaling genes, and many other genes that respond to abiotic stresses [99]. The results obtained from microarray analysis revealed that only a few genes were affected under salt stress in *Thellungiella salsuginea* compared to *Arabidopsis* [100]. In another study, it was observed that in *Thellungiella salsuginea*, about 154 genes were identified as compared to *Arabidopsis* under various stresses [101]. A diagram illustrating salt stress tolerance mechanisms in halophytes is shown in Figure 5.

*Arabidopsis* and *Lepidium crassifolium* have been studied as model plants to contrast a glycophyte with a halophyte, respectively, and to identify genes involved in oxidative and osmotic stress tolerance. Independent transgenic lines expressing random genes from *L. crassifolium* in *Arabidopsis thaliana* enhanced plant salinity tolerance [102]. Other studies were conducted with *Salicornia brachiata*, which grows in saline marshes under extreme abiotic stresses. This plant is considered to be an efficient source of stress response genes and promoters as it has the potential to grow under adverse environmental conditions [97]. Salinity-resistant transgenic plants including *Jatropha*, Cumin, and Castor were developed using salt-responsive genes isolated from *Salicornia brachiata* [103]. *Salicornia* species are considered functional foods as they contain metabolites and sulfur-rich seed storage proteins [104]. *Porteresia coarctata* is a wild halophyte that has the ability to grow in extreme saline soils. Around 152,367 unique transcript sequences were identified; 15,158 of these genes are related to salinity and submerged tolerance and the analysis of these genes will help unravel the key metabolic pathways involved in tolerance. These genes can also be utilized to introduce salinity and submerged tolerance traits in rice [105]. Table 3 shows the salt-responsive genes present in halophytes and recipient plants that express the genes.

## 7. Promoters for Salt-Responsive Halophytic Genes

A strong promoter is needed for the genetic engineering of plant crops to attain the desired level of transgene expression. In *T. halophila*, stress-related genes are expressed constitutively as compared to *A. thaliana* where they are not [123]. This study revealed that in halophytes, a transcriptional regulatory network for stress-responsive genes is fully functional. Another study was carried out in halophytes that led to the identification of *cis*-regulatory elements of stress-responsive genes and stress-inducible motifs [124]. The promoter of the *AcBADH* gene from *Atriplex centralasiatica* contains two salt-responsive enhancer regions and one silencer region. The enhancer regions are located from −1115 to −890 and −462 to −230, while the silencer region is from −890 to −462. The *AcBADH* promoter is strongly induced by salinity stress [125].

Another strong and salt-inducible promoter is *SIBADH;* the promoter fragment (-300 bp) was identified in *Suaeda liaotungensis.* This promoter supported a 6.3-fold higher expression under salinity in contrast to non-stressed conditions [106]. To measure expression levels, GUS is widely used as a reporter gene in transformation in microorganisms and animals. It is found in various bacterial species such as *Shigella, Bacteroides* and *Clostridium, S. liaotungensis,* and *Salicornia europaea* that contain CMO genes [120]. GUS showed increased expression in transgenic *Arabidopsis*, while a halophyte *T. halophila* contains a *TsVP1* gene promoter having a 130 bp specific *cis*-acting element responsive to salt stress. The *SIPEAMT* gene of *S. liaotungensis* with an 897 bp promoter region also showed an 18.6-fold increase in the beta-glucuronidase (GUS) activity under 200 mM NaCl stress [126]. They determined that even a small portion of the promoter contains a *cis-*acting element that allows regulation of gene expression under salinity stress. A choline monooxygenase (CMO) gene was found in Chenopodiaceae and Amaranthaceae. In plants, usually, the activity of CMO is low and unstable but it can be a critical rate-limiting step in the biosynthesis of glycine betaine [127]. Promoters of these genes are inducible under salinity stress. Crassulacean acid metabolism (CAM) genes were studied in *M. crystallinum,* and the transcriptional activation of salt-responsive genes occurs due to the enhancer and silencer regions of the gene promoter [128]. CAM genes were found in various plant species. In model plant species such as *Arabidopsis* and rice, it was revealed that CAM proteins are encoded by gene families. These genes play a significant role in the regulation of growth, development, and abiotic stress resistance in plants [129].

A tissue-specific promoter *AISAP* was examined in *Aeluropus littoralis* [130]. It was found that the expression level of a *gusA* fusion with this promoter was the same in transgenic rice under the control of the *AISAP* gene as in *A. littoralis* [131]. *AISAP* and *OsSAP9* are two orthologs of the regulatory region of the promoter and provide the basis for variation in regulation specificity and stress induction in rice. The *TsVP1* gene from the halophyte *T. halophile* contains a 130 bp *cis-*acting element in the promoter region of vacuolar H^+^-pyrophosphatase. It helps to enhance GUS fusion expression under salinity stress in transgenic *Arabidopsis* [132]. Under the conditions of biotic and abiotic stresses, the expression of the reporter gene can be controlled by the *CBL1* gene promoter obtained from *Ammopiptanthus mongolicust* [133]. The enhancer and repressor binding sites in the *cis-*regulatory region were also found in the *SbpAPX* gene. This gene was found in *S. brachiata* [134]. The salt stress-responsive *cis-*regulatory motifs were present in the *SbGSTU* promoter. In *S. brachiata,* these motifs regulate the expression of the *GSTU* gene [124]. Thus, halophytes can be utilized as a source of genes for engineering abiotic stress tolerance in crops. Table 4 shows the details of promoters used to stimulate salinity resistance traits in plants.

## 8. Transgenic Approach for Engineered Plants Having Enhanced Salt Tolerance

**Figure 6:**
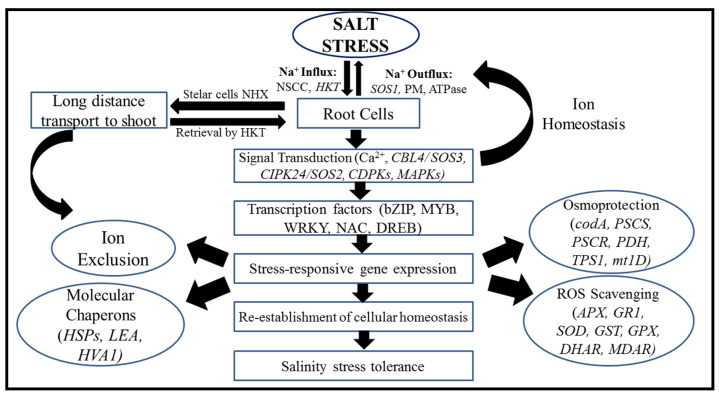
Factors involved in enhanced plant salt tolerance.

It was reported by Rao et al. [53] that for salt tolerance in plants, the breeding strategy is not particularly recommended due to reproductive restrictions and there is a high probability for the transfer of undesirable traits. Genetic engineering is considered to be suitable for single gene transfer [58]. Plants have been developed from a single plant cell by the direct transfer of the gene of interest into the genome to create transgenic plants. The use of genetic engineering techniques involves traits such as resistance to pesticides, pests, better nutritional value, and improved product shelf life, which can contribute to improvements in sustainable agriculture [135]. Figure 6 shows the factors involved in enhanced salt tolerance in plants. The transgenic approach is also utilized to enhance the resistance to abiotic stress in plants [136]. Table 5 summarizes reports on gene transfer into target plants for enhanced salt tolerance.

## 9. Development of Salt Tolerant Glycophytes using Halophytic Salt Tolerance Genes

Plants are classified into two groups: salt-tolerant halophytes and salt-sensitive glycophytes depending on their growth ability in saline environments. Halophytes have the ability to grow in a saline environment including coastal marshes and inland deserts. Monocot halophytes have the potential to achieve optimum growth at less than 50 mM NaCl while in the case of dicot halophytes they can grow at approximately 100–200 mM salt [151]. Glycophytes are highly affected by saline habitats and do not grow well at 100-200 mM NaCl [92]. Various studies were conducted to identify salt tolerance genes in halophytes [96]. Over the last several years, genetic engineering has been commonly used to introduce salt tolerance in glycophytes by the transfer of salt tolerance responsive genes from halophytes [152]. For this purpose, different plants are used as model plants for the introduction of salinity tolerance genes. *Arabidopsis,* tobacco, and many other crop plants have been used to enhance ion homeostasis and salt tolerance [73].

**Figure 7:**
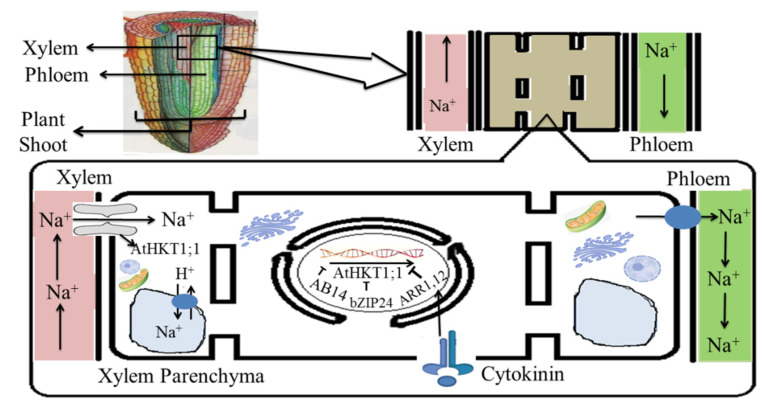
Model showing *AtHKT1;1* and the negative regulators of *AtHKT1;1* expression.

The most commonly monitored phenomena related to salinity are the dispersion of sodium ions in vacuoles, sodium ion efflux, and the prevention of sodium ion influx by the antiporter [153]. Many antiporters have been characterized functionally after their isolation from glycophytes and halophytes. It was observed that some of the glycophytic transporters that are encoded by the *NHX, ATPase, SOS*, and *HKT* genes led to salt tolerance in the range of 150–250 mM NaCl when expressed under the control of the constitutive CaMV35S promoter in transgenic plants [154]. For evolving salt tolerance in various crops such as tomato, maize, brassica, and wheat, constitutive expression of the glycophytic *NHX* gene obtained from *Arabidopsis thaliana* was used [155]. However, other genes such as *BnNHX1 (Brassica napus), HbNHX1(Hordeum brevisubulatum),* and *GhNHX1(Gossypium hirsutum)* were used to develop salt tolerance in tobacco. It was reported that salt tolerance was conferred by the *NHX1* gene obtained from both halophyte and glycophytes, although they differ in the level of salt tolerance. A 75% amino acid sequence similarity was observed for the antiporter *Ag*NHX1 from *Atriplex 15melina* and *At*NHX1 from *Arabidopsis thaliana.* As compared to glycophytes, transgenic plants overexpressing *AgNHX1, SaNHX1*, or the *SsNHX1* gene showed up to 300–400 mM NaCl tolerance [156]. In genetically modified tobacco plants, upregulation of the *SbNHX1* gene showed salt tolerance up to 200 mM NaCl while in transgenic jatropha and castor plants, salt tolerance was observed up to only 100 mM NaCl [157]. However, in transgenic plants, the upregulation of other halophytic genes such as *SbpAPX, SbUSP,* and *SbGSTU* also showed salt tolerance up to 200–300 mM NaCl [158]. The *TIP1* gene obtained from the halophyte *T. salsuginea* revealed enhanced salt tolerance in transgenic *Arabidopsis* plants as compared to the glycophyte *Panax ginseng* [159]. Genes from rice such as APX and GST showed tolerance of up to 150–200 mM NaCl when compared to similar genes obtained from the halophyte *S. brachiata* in transgenic plants [160]. Different levels of abiotic stress tolerance in rice, tobacco, and wheat were observed by up-regulation of the stress-associated protein *AISAP* from *Allocasuarina littoralis* [131]. It was reported that many abiotic stresses induced *AISAP* transcripts but the rice gene *OsSAP9* is also influenced by cold and heat treatments. The negative regulators of *AtHKT1; 1* expression are shown in Figure 7.

The antiporter SOS1 gene and its over-expression were studied in *Thellungiella* and contrasted to expression in *Arabidopsis* [161]. In *Thellungiella*, *SOS2, NHX1,* and *HKT1*, which are involved in sodium exclusion and compartmentation, have been expressed at higher levels [123]. Halophytes can serve as model plants to discover different stress-responsive genes for enhancing the salt resistance of glycophytes to allow cultivation in saline and arid areas for sustainable agriculture.

## 10. MicroRNAs (miRNA), a New Target for Improving Plant Tolerance to Salt Stress

Microarray and high throughput deep sequencing methods are used to identify plant miRNAs induced under salinity, as presented in Table 6. It was observed that plant miRNAs are present in all of the main plant parts such as the leaf, root, stem, and flower (Table 5). It was reported by Fu et al. [162] that among all the crops, the highest numbers of miRNAs have been identified in *Zea mays* (1077 miRNAs). *Mesembryanthemum crystallinum*, *Medicago truncatula*, *Vicia faba*, and *Ipomoea batatas* contain 882, 876, 693, and 650 miRNAs, respectively, under saline conditions [163]. The numbers of miRNAs in plants may vary and depend on plant species, tissue specificity, development stages, and intensity of salinity stress. The degree of salt stress may up- and down-regulate the expression level of miRNAs in plants. Jodder [164] observed that the expression of miR167 in oat panicles is negatively associated with an increase in the degree of salt stress. With a 0.25% increase in NaCl, miR156, miR157, and miR172 are up-regulated in cotton, and the expression decreases with a further increase in salt concentration. Nanoparticles have the ability to affect the expression level of plant miRNAs [165].

The expression levels of miRNAs highly depend on plant developmental stages, as it was reported that few miRNAs are expressed in early growth under saline conditions while others appear in the late stages. It was observed by Luan et al. [178] that zma-miR169 shows initial up-regulation and then down-regulation under salinity stress. In cotton, miRNAs and their targets such as miR156-SPL2, miR159-TCP3, miR162-DCL1, miR395-APS1, and miR396-GRF1 show a negative correlation of expression levels [165]. Some of the miRNAs are induced under salt stress such as miR156, miR319, and miR528 while miR164 and miR397 are repressed [179]. The degree of salinity stress may increase or decrease the expression level of some miRNAs in plants. For instance, in *Arabidopsis thaliana, Triticum aestivum,* and *Agrostis stolonifera,* the expression level of miR393 increases under salt stress. However, in contrast, the expression level of miR393 decreases in *Oryza sativa*, *Gossypium* sp., and *Spartina alterniflora* under similar conditions [180]. The expression level of some miRNAs such as miR167, miR390, miR402, and miR414 have been observed only in a few plant species under salinity stress.

Glycophytes cannot tolerate high salinity levels while halophytes can tolerate and survive at up to 1000 mM NaCl. Glycophyte plants may have the potential to adapt a salt tolerance mechanism by following various strategies of gene regulation used by halophytes. The role of halophyte miRNAs may follow various strategies of salinity resistance improvements in crops by incorporating them in genetic engineering and plant selection programs. For instance, Gharat [173] observed that the expression of *Suaeda maritima* sma-miR2 and sma-miR5 increases in seawater, suggesting that their metabolic regulatory roles are restricted to saline environments. About 246 miRNAs have been identified in *E. salsugineum.* A significant response to salt stress in *E. salsugineum* was observed by the expression of 26 conserved miRNAs and four novel miRNAs [181]. Seedlings of *M. crystallinum* were treated with 200mM NaCl and it was observed that 135 conserved miRNAs and the hairpin precursor of 12 novel mcr-miRNAs were expressed [182]. In another example, *Halostachys capsica*, a salt tolerant shrub, was treated with salt and it was observed that 31 conserved miRNAs and 12 novel miRNAs were up-regulated while 48 conserved miRNAs and 13 novel miRNAs were down-regulated by salinity stress in *H. caspica* [183].

## 11. Conclusions and Future Perspectives

Plants integrate cellular, physiological, and molecular responses for salt stress tolerance. Various studies have been carried out on the plant salt resistance mechanisms that control ion homeostasis, osmoregulation, ROS detoxification, hormone metabolism, and stress signaling, but there is still a lack of information from genomic, transcriptomic, and proteomic studies. Genetic engineering of salt tolerance in plants has great potential. However, the continuous release of nanoparticles into the surrounding soil may affect plant growth and development. Nanoparticles may alter seed germination as well as various stages of crop production. Various kinds of nanoparticles have been found in the environment such as ZnO, CuO, TiO2, and Fe3O4. All of these nanoparticles showed some positive and negative results against seed germination, root and shoot growth, biomass production, and physiological as well as biochemical activities. These nanoparticles become adsorbed onto the plant surface and are transported to different plant tissues. However, the low concentration of nanoparticles does not show any negative effect on plants and appears to be beneficial for their growth and developmental process. Higher concentrations of nanoparticles cause toxicity by ROS which leads to the disruption of the cellular membrane. It has been considered that some nanoparticles could replace the use of toxic chemicals and fertilizers in the near future. Still, further research needs to be carried out to analyze the effect of nanoparticles on plants and the surrounding environment.

Progress has been made in developing salt-tolerant cultivars, but there are still many questions related to salt stress tolerance in plants that need to be addressed with the help of molecular marker development for gene mapping, EST library development, and integration of complete genome sequences for Arabidopsis, rice, and maize. High throughput sequencing is the most powerful technology for the identification of salt stress-responsive miRNAs. After the identification of miRNAs in plants, there is still an empty space that needs to be filled for the analysis of function carried out for salt tolerance improvement through miRNA manipulation in crops. Applications of nanoparticles will play a significant role in the modification of salt-tolerance genes in plants. Many advanced strategies have been used to date to modulate genes in plants under salinity stress, including nanoparticle transport across the plant cell and chloroplast membranes to target their genetic makeup. In addition, miRNAs can be introduced to develop salt resistance in crops. The over-expression and knocking down of miRNAs may promote the development of salinity resistance in transgenic plants.

The various modifications in small RNA sequencing technologies and analysis of miRNAs will be important for the development and growth of salt-tolerant cultivars. Many advances in genomics and metabolomics analyses of crop plants may improve the resolution of complex networks and unravel the mechanism(s) of abiotic stress tolerance. It will be essential to identify candidate gene(s) that have the ability to confer stress tolerance in plants without affecting growth and yield. It has been observed that conventional breeding methods did not significantly improve salt stress tolerance in plants and in addition, the crossing method reduced crop yield. In order to develop salt-tolerant plants, some points need to be addressed such as (i) how under the unstable natural environment genetically modified plants respond to soil salinity conditions, (ii) how soil salinity affects the transgenic plants at different growth stages including seed germination and reproductive stage, (iii) the effects of transgene expression on plant growth and development as well as nitrogen use efficiency (NUE) under saline conditions, (iv) yield of transgenic plants, and (v) disease resistance. Additional research still needs to be performed to understand stress perception, signaling, transcription factors, and genes associated with the salinity stress response.

## Figures and Tables

**Table 1:** List of plant species and their secondary metabolites [50].

Fodder Crops	Secondary Metabolites
*Atriplex nummularia*	Saponin, Alkaloids, Tannins, Nitrate
*Atriplex leucoclada*	Saponin, Alkaloids, Tannins
*Atriplex halimus*	Saponin, Flavonoids, Alkaloids, Tannins, Nitrate
*Diplache fusca*	Flavonoids, Alkaloids
*Halocnemum strobilecum*	Saponin, Flavonoids, Alkaloids, Tannins, Nitrate
*Haloxylon salicornicum*	Saponin, Flavonoids, Alkaloids, Tannins
*Kochia eriophora*	Alkaloids, Tannins
*Juncus acutus*	Flavonoids, Alkaloids, Tannins, Nitrate
*Juncus arabicus*	Alkaloids, Tannins
*Juncus subulatus*	Alkaloids, Tannins, Flavonoids
*Limonium pruinosum*	Saponin, Alkaloids, Tannins
*Nitraria retusa*	Saponin, Tannins
*Salsola glauca*	Saponin, Flavonoids, Alkaloids
*Suaeda fruticosa*	Alkaloids, Tannins, Nitrate
*Tamarix aphylla*	Saponin, Tannins
*Salsola tetrandra*	Nitrate
*Tamarix mannifera*	Saponin, Tannins
*Zygophyllum album*	Saponin, Flavonoids, Alkaloids, Tannins, Nitrate
*Sesbania sesban*	Saponin, Alkaloids

**Table 2:** Salt responsive genes in various plant species along with their respective gene functions.

Gene Name	Plants	Gene Functions	References
*SOS1,SOS2,* *AtNHX1*	*Brassica campestris Brassica juncea*	Na^+^/K^+^ plasma membrane antiporter, calcium-binding protein, and protein kinase	[65]
*AtSKIP*	*Arabidopsis thaliana*	Transcription factor, splicing, and polyadenylation	[66]
*OsHsp17.0* *OsHsp23.7*	*Oryza sativa* L.	Transporting proteins and heat-shock proteins	[67]
*DcHsp17.7*	*Daucus carota*	Cell viability and membrane stability under heat stress	[68]
*JcDREB*	*Arabidopsis thaliana*	Transcription factor	[69]
*AtNHX1*	*Arabidopsis thaliana*	Calcium-binding protein, vacuolar Na^+^/K^+^ antiporter	[70]
*OsRab7*	*Oryza sativa* L.	Vesicle trafficking gene enhanced growth and proline	[71]
*PeXTH*	*Populus euphratica*	Higher cell viability, water holding capacity, and membrane integrity	[72]
*CYP94*	*Oryza sativa*	Enhanced *CYP94C2b* expression	[73]
*SOS1*	*Nicotina tabacum*	Plasma membrane, Na^+^/K^+^ and vacuolar Na^+^/K^+^ antiporter	[74]
*mtlD*	*Escherichia coli*	Higher mannitol 1 phosphate dehydrogenase levels	[75]
*TaSTRG*	*Triticum aestivum*	Increase salinity and water deficit resistance	[76]
*AtSTO1*	*Arabidopsis thaliana*	Higher root, pith size, and photosynthesis	[77]

**Table 3:** Salt-responsive genes present in halophytes and recipient plants.

Halophytes	Genes	Description	Recipient Plants	References
*Aeluropus littoralis*	*AlNHX1*	Vacuolar Na^+^/H^+^ antiporter	*Nicotiana tabacum*	[106]
*Atriplex hortensis*	*AhBADH*	Glycine betaine synthesis	*Solanum lycopersicum*	[107]
*Avicennia marina*	*AmMDHAR*	ROS scavenging	*Nicotiana tabacum*	[108]
*Salicornia brachiata*	*SbASR1*	Ascorbate regeneration and ROS scavenging	*Nicotiana tabacum*	[108]
*Salicornia brachiata*	*SbpAPX*	Peroxisomal ascorbate peroxidase	*Nicotiana tabacum*	[109]
*Salicornia brachiata*	*SbpAPX*	Peroxisomal ascorbate peroxidase	*Arachis hypogea*	[110]
*Salsola soda*	*SsNHX1*	Vacuolar Na^+^/H^+^ antiporter	*Alfalfa*	[111]
*Suaeda liaotungensis*	*SlBADH*	Glycine betaine synthesis	*Zea mays*	[112]
*Suaeda salsa*	*SsCAX1*	Vacuolar H^+^/Ca^2+^ transporter	*Arabidopsis*	[113]
*Suaeda salsa*	*SsGST*	Glutathione S-transferase	*Oryza sativa*	[114]
*Suaeda salsa*	*SsVP*	Vacuolar-H^+^-pyrophosphatase	*Arabidopsis*	[115]
*Thellungiella halophila*	*ThSOS1*	Salt overly sensitive gene	*Arabidopsis*	[116]
*Thellungiella salsuginea*	*TsTIP1*	Tonoplast *AQP* gene	*Arabidopsis*	[117]
*Tamarix androssowii*	*TaMnSOD*	Manganese superoxide dismutase	*Populus*	[118]
*Spartina alterniflora*	*SaVHAc1*	Vacuolar H^+^-ATPase subunit Cl	*Oryza sativa*	[119]
*Salicornia europaea*	*SeCMO*	Enhanced glycine betaine synthesis	*Nicotiana tabacum*	[120]
*Kalidium foliatum*	*V-ATPase*	Vacuolar-H^+^-pyrophosphatase	*Arabidopsis*	[121]
*Atriplex gmelini*	*AgNHX1*	Vacuolar Na^+^/H^+^ antiporter	*Oryza sativa*	[122]

**Table 4:** Promoters used to improve salt tolerance traits in crop plants [58].

Transgene	Gene Isolated	Promoters	Transgenic Crop
Ion exclusion Na^+^/H^+^ antiporter (*SOS1)*	*Arabidopsis*	Constitutive	*Nicotiana tabacum*
Na^+^/H^+^ antiporter (*SOD2)*	*Salicornia brachiata*	Stress inducible	*Oryza sativa*
Tissue tolerance Na^+^/H^+^ antiporter (*NHX*)	*Arabidopsis*	Constitutive	*Fagopyrum esculentum*
Tissue tolerance Trehalose-6-phosphate synthase (*TPS*)	Yeast	Constitutive	*Medicago sativa*
Tissue tolerance Trehalose-6-phosphate phosphatase (*TPP*)	Rice	Stress inducible	*Solanum lycopersicum*
Mannitol-1-phosphate dehydrogenase (*mt1D*)	*E.coli*	Shoot expression	*Oryza sativa*
Myoinositol O-methyltransferase	*M. crystallinum*	Constitutive	*Triticum aestivum*
Tissue tolerance Ascorbate (*APX*)	*Arabidopsis*	Constitutive	*Nicotiana tabacum*
Glutathione S-transferase (*GST*)	Tomato	Protein targeted to chloroplast/cytosol	*Oryza sativa*
Mitogen activated protein kinase (*MAPK*)	Chickpea	Constitutive	*Nicotiana tabacum*
Sucrose protein kinase	Rice	Inducible	*Oryza sativa*
Transcription factors *DREB*	*Pennisetum glaucum*	Constitutive & inducible	*Nicotiana tabacum*

**Table 5:** Gene transfer into target plants for enhanced salt tolerance.

Desired Gene	Donor Plant	Target Plant	References
*codA*	*Arthrobacter globiformis*	*Solanum lycopersicum*	[137]
*Cox*	*Arthrobacter pascens*	*Oryza sativa*	[138]
*TPS1*	Yeast	*Solanum lycopersicum*	[139]
*AtTPS1*	*Arabidopsis*	*Nicotiana tabacum*	[140]
*mtID*	*Triticum aestivum*	*Escherichia coli*	[141]
*S6PDH*	*Malus domestica*	*Japanese Persimmon*	[142]
*P5CS*	*Vigna acontifolia*	*Nicotiana tabacum*	[143]
*nhaA*	*E.coli*	*Arabidopsis*	[141]
*AtNHX1*	*Arabidopsis*	*Solanum lycopersicum*	[144]
*BnNHX1*	Brassica	*Nicotiana tabacum*	[145]
*GlyII*	*Oryza sativa*	*Nicotiana tabacum*	[146]
*GmbZIP1*	Soybean	*Arabidopsis, Nicotiana tabacum*	[147]
*BrERF4*	Brassica	*Arabidopsis*	[148]
*T30hsp70*	*Trichoderma harzianum*	*Arabidopsis*	[149]
*GhMPK2*	Cotton	*Nicotiana tabacum*	[150]

**Table 6:** Numbers of salt-responsive miRNAs identified under salt stress at varying concentrations of NaCl.

Plants	NaCl Concentration	miRNA Number	References
*Arabidopsis thaliana*	150 mM	118	[166]
*Glycine max*	125 mM	238	[167]
*Leymus chinensis*	100 mM	148	[168]
*Medicago truncatula*	20 mM	876	[169]
Musa nana	300 mM	181	[170]
*Oryza sativa*	200 mM	498	[171]
*Panicum virgatum*	0.5 %	273	[172]
*Suaeda maritima*	255 mM	147	[173]
Zea mays	250 mM	1077	[174]
*Vicia faba*	150 mM	693	[175]
*Thellungiella salsugniea*	200 mM	246	[176]
*Raphanus sativus*	200 mM	204	[177]

## Data Availability

Not applicable.
